# Shift detection discrepancy between ExacTrac Dynamic system and cone‐beam computed tomography

**DOI:** 10.1002/acm2.13567

**Published:** 2022-02-21

**Authors:** Vivian U. Y. Chow, Michael L. M. Cheung, Monica W. K. Kan, Anthony T. C. Chan

**Affiliations:** ^1^ Department of Clinical Oncology Prince of Wales Hospital Hong Kong SAR China; ^2^ Department of Clinical Oncology The Chinese University of Hong Kong Hong Kong SAR China

**Keywords:** cone‐beam computed tomography, optical surface tracking, patient positioning, stereotactic radiosurgery, thermal mapping

## Abstract

Accurate detection of patient shift is essential during radiation therapy such that optimal dose is delivered to the tumor while minimizing radiation to surrounding normal tissues. The shift detectability of a newly developed optical surface and thermal tracking system, which was known as ExacTrac Dynamic (EXTD), was evaluated by comparing its performance with the image guidance under cone‐beam computed tomography (CBCT). Anthropomorphic cranial and pelvis phantoms with internal bone‐like structures and external heat pad were utilized to study the shift detection discrepancy between EXTD system and CBCT. Random displacements within the range of ± 2 cm for translations and ± 2 degrees for rotations were intentionally applied to the phantom. Positional shifts detected by optical surface and thermal tracking (EXTD_Thml), stereoscopic X‐ray (EXTD_Xray), and CBCT were compared in 6 degrees of freedom. The translational difference between EXTD_Thml and CBCT was 0.57 ± 0.41 mm and 0.66 ± 0.40 mm for cranial and pelvis phantom, respectively, while it was 0.60 ± 0.43 mm and 0.76 ± 0.49 mm between EXTD_Xray and CBCT, respectively. For rotational movement, the difference between EXTD_Thml and CBCT was 0.19 ± 0.16° and 0.19 ± 0.22° for cranial and pelvis phantom, respectively, while it was 0.13 ± 0.18° and 0.65 ± 0.46° between EXTD_Xray and CBCT, respectively. This study demonstrated that the EXTD system with thermal mapping ability could offer comparable accuracy for shift detection with CBCT on both cranial and pelvis phantoms.

## 1 INTRODUCTION

Stereotactic radiosurgery (SRS) is used to treat intracranial lesions through non‐coplanar isocentric arcs in single fraction.^1^, ^2^ Since the treatment is delivered with a high dose in single fraction, rigid patient immobilization and positional accuracy are particularly important for SRS. SRS was originally developed with a frame‐based head fixation for rigid patient immobilization. However, it is invasive and patients may feel discomfort.^3^ These drawbacks facilitated the shift from a frame‐based approach to frameless stereotactic treatment, which requires stringent image guidance for monitoring and verifying patient positions.^4^, ^5^ Image guidance is also essential for stereotactic body radiation therapy (SBRT) for accurate target localization such that a smaller setup margin can be used to reduce the volume of normal tissues irradiated while delivering a high dose to the tumor. On‐board cone‐beam computed tomography (CBCT) is one of the commonly used modalities of image guidance, which can provide three‐dimensional (3D) anatomical images under image‐guided radiation therapy (IGRT).^6^, ^7^, ^8^ However, CBCT images cannot be acquired during treatment delivery and hence hindering intrafractional motion monitoring. Also, the relatively long acquisition time and high radiation dose to patient limit the clinical use of CBCT.^9^, ^10^, ^11^, ^12^

Surface‐guided radiation therapy (SGRT) using real‐time optical surface tracking has therefore been developed recently to complement IGRT for real‐time intrafractional patient monitoring.^13^, ^14^, ^15^, ^16^ Such a system is usually composed of a projector and one or several cameras to acquire live 3D surfaces of the patients.^17^ Through comparing the live surfaces and the reference surface, the system can calculate the geometric shifts of patient positions in both translational and rotational directions. Also, an additional level of safety and accuracy can be guaranteed during the treatment delivery through automatic beam‐hold if the deviation of live and reference surface exceeds the preset tolerance. Therefore, it has the potential to improve the clinical outcomes with accurate target localization and irradiation.^18^

Structured light scanning (SLS) is the core technology of SGRT and is currently been applied in several commercial optical surface tracking systems such as AlignRT (Vision RT, London, UK), optical surface monitoring system (OSMS) (Varian, Palo Alto, CA), Catalyst (C‐RAD, Uppsala, Sweden) and Identify (Varian).^17^, ^19^, ^20^, ^21^ Although SLS is used commercially, there are still several limitations of the usage of SLS in radiotherapy. One of the limitations is the misalignment of live surface and reference surface for objects with translational or rotational symmetries using the iterative closest point algorithm, which is known as registration sliding effects.^22^ Also, SLS is based on the assumption that a good correlation exists between the surface and internal organs. This assumption is highly dependent on the treatment sites and for some easily deformed body regions such as facial tissues, further IGRT verification is required for accurate treatment delivery.^22^ However, current SLS systems do not equip with an X‐ray imaging system that is independent of LINAC and hence hinder intrafractional monitoring.

Recently, a newly developed optical surface and thermal tracking system known as ExacTrac Dynamic (EXTD) (BrainLab AG, Munich, Germany) has been released for clinical use, which claimed that the above‐mentioned limitations could be overcome by the combination of stereoscopic X‐ray, optical surface, and thermal tracking in a single system.^18^, ^22^ It is a consolidation of IGRT and SGRT for both interfractional and intrafractional motion monitoring. IGRT verification is achieved by using two X‐ray tubes mounted on the floor next to the LINAC and two flat‐panel detectors mounted on the ceiling.^18^ At the same time, the use of an optical surface tracking system together with thermal signals from a ceiling‐mounted thermal camera might reduce the registration sliding effects for SGRT.

Previous studies were conducted to evaluate the performance of a pure optical surface tracking system using a phantom approach. However, no study has been conducted to evaluate the positional accuracy provided by the newly released EXTD. This study, therefore, aims to evaluate the performance of optical surface and thermal tracking (EXTD_Thml) and stereoscopic X‐ray (EXTD_Xray) equipped in EXTD for shift detection compared with CBCT using anthropomorphic cranial and pelvis phantoms.

## 2 METHODS

### 2.1 System description

The TrueBeamSTx LINAC (Varian) installed at the Prince of Wales Hospital is equipped with 120 high‐definition multi‐leaf collimator (Varian), the integrated kilovoltage (kV) on‐board imaging (OBI) (Varian) that enables the generation of CBCT images, and 6 degrees of freedom (DOFs) robotic couch known as PerfectPitch (Varian). During CBCT, the X‐ray tube of the OBI rotates around the patient to obtain multiple planar images for 3D image reconstruction. Automatic 3D‐3D matching between the acquired CBCT image and the reference computed tomography (CT) image is conducted to obtain a set of geometric shifts of the couch such that their matching is optimal. The shifts obtained are then applied to remotely correct the couch position from the control room.

**FIGURE 1:**
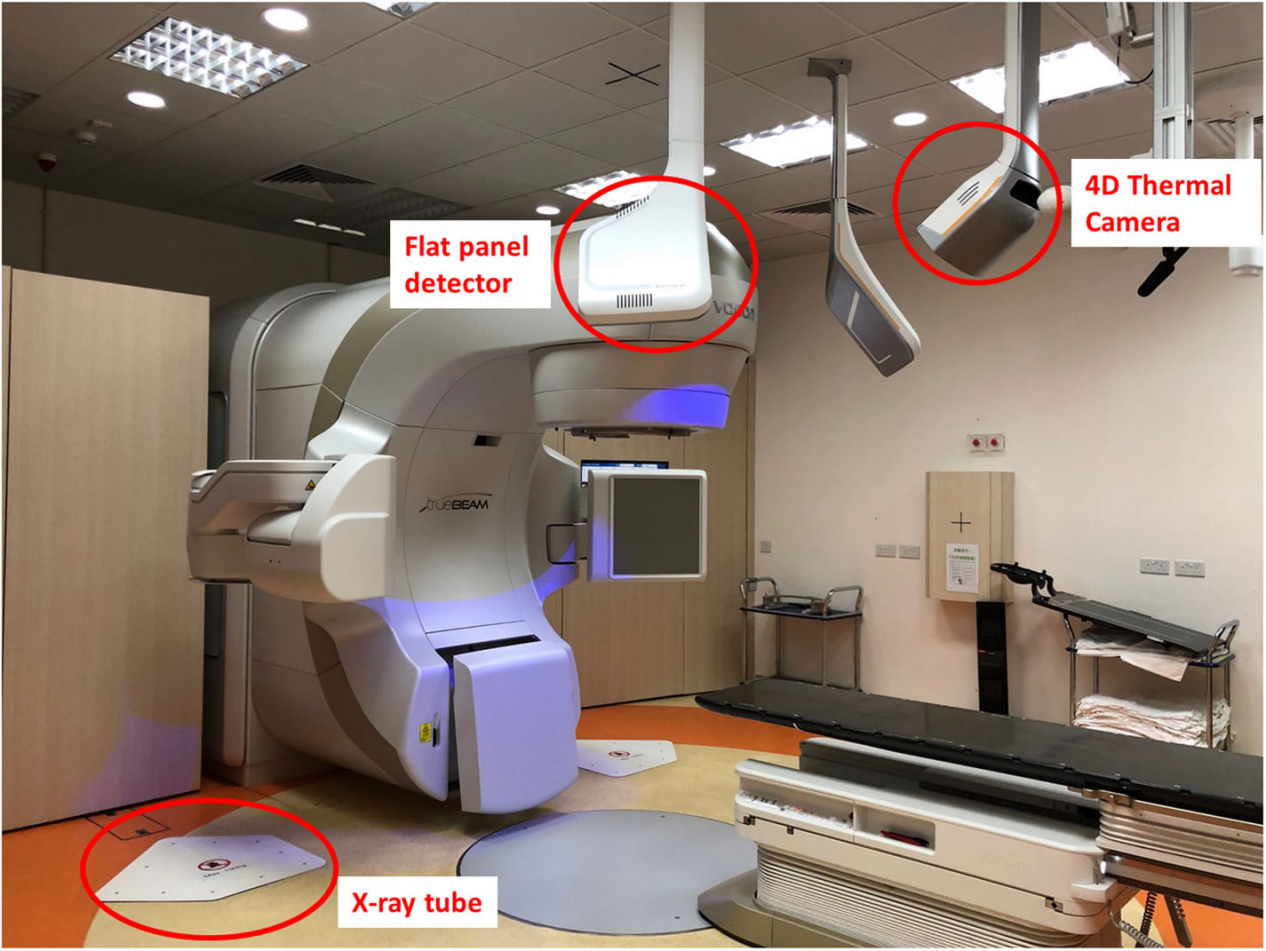
Components of ExacTrac Dynamic (EXTD) system It consists of two x‐ray tubes on the floor with two flat panel detectors on each side of a set of optical & thermal cameras that is centrally ceiling‐mounted above the couch.

The LINAC is also equipped with the newly released EXTD system, which is composed of two X‐ray tubes in the floor boxes with two flat‐panel detectors on each side of a set of optical and thermal cameras that is centrally ceiling‐mounted above the couch as shown in Figure 1. During treatment, EXTD is used to monitor patient position using the optical surface and thermal camera. Once the patient's position is out of the preset tolerance, beam‐hold will be triggered and a stereoscopic X‐ray will be used for further verification. The stereoscopic X‐ray acquired will be fused with the digitally reconstructed radiographs (DRRs) obtained from planning CT using either manual or automatic matching tools. Once the desired match between X‐ray and DRRs is achieved, the shift in 6 DOFs can be transferred to the couch remotely to correct the positioning. Before the measurement, the EXTD was calibrated to the LINAC coordinate system using the System Calibration Phantom (BrainLab AG) with eight X‐ray markers inside.^23^ First, the engraved lines on the surface of the phantom were aligned precisely with the treatment room lasers, followed by the acquisition of two oblique X‐ray images using EXTD. The system detected the X‐ray markers positions and calculated the calibration to the LINAC isocenter using the pattern of the known marker.^23^ Simultaneously, the optical and thermal camera registered the known geometry of the phantom, which had been positioned at the LINAC isocenter. After that, a Winston–Lutz pointer with a metal sphere was placed at the LINAC isocenter. The EXTD detected the sphere position and calculated the offset of the EXTD isocenter calibration from the LINAC isocenter. The offset should be less than 1 mm in 3D for a successful isocenter calibration.

### 2.2 Phantom‐based experiments

Anthropomorphic cranial and pelvis verification phantoms (BrainLab AG) incorporated with bone‐like structures were used in this study. The cranial phantom immobilized with Brainlab cranial 4Pi open face mask and pelvis verification phantom were scanned on Brilliance CT big bore scanner (Philips, Amsterdam, the Netherlands). The CT slices with thickness 0.5 mm were then imported to the iPlan treatment planning system (BrainLab AG) to prepare for a treatment plan with an isocenter defined in the phantom.

**FIGURE 2:**
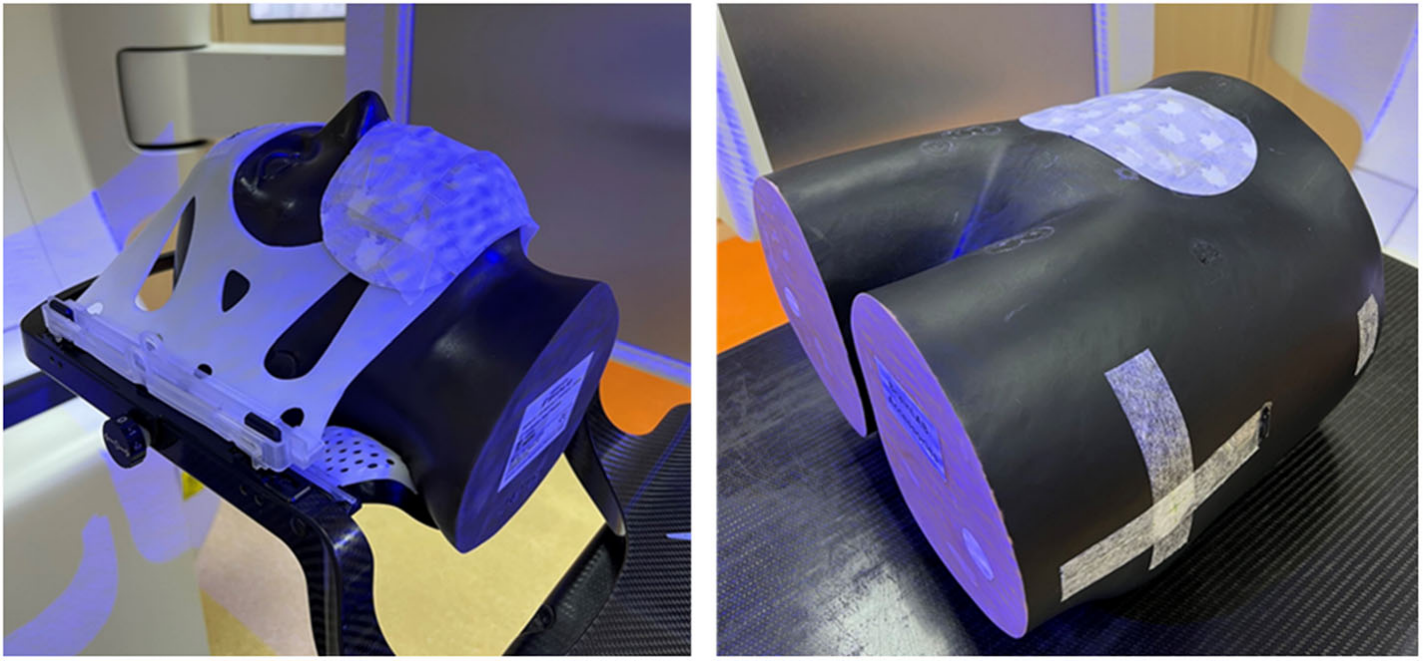
Anthropomorphic cranial (left) and pelvis (right) verification phantoms. The cranial phantom was fitted with Brainlab cranial 4Pi open face mask. Heat pad was applied on the phantom to generate temperature difference for thermal tracking.

To generate temperature difference for thermal tracking, heat pads were applied on the phantoms in our study as shown in Figure 2. First, the phantom was pre‐positioned to the isocenter with the EXTD, followed by final positioning with the couch shift proposed by CBCT. EXTD then updated the final positioning with the acquisition of both reference optical and thermal signature. The current positioning reported by EXTD_Thml, EXTD_Xray, and CBCT was recorded as initial reading. After that, random linear movements within ±2 cm and rotational movements within ±2 degree were applied to the phantom. The real‐time positioning reported by the optical surface and thermal tracking system was recorded as the final reading of EXTD_Thml. The phantom was then imaged with two oblique X‐rays in EXTD and these two images were registered with corresponding DRRs from planning CT to calculate a geometric shift in 6 DOFs, which was recorded as the final reading of EXTD_Xray. Then, CBCT was used to scan the phantom and the acquired images were matched with planning CT using six‐dimensional (6D) image registration to generate the final reading of CBCT. The differences between the initial and final reading were the shifts reported by EXTD_Thml, EXTD_Xray, and CBCT. For simplicity, the shift detectability of EXTD_Thml and EXTD_Xray relative to CBCT in 6 DOFs was also summarized into two components: Vector_translation_ and Vector_rotation_ by Equations (1) and (2). The above procedure was repeated 20 times for both cranial and pelvis verification phantoms with a complete reposition of the phantom to the planned treatment position before the application of random shifts.

(1) $$\mathrm{Vecto}r_{\mathrm{translation}}=\sqrt{\Delta_{\mathrm{vertical}}^{2}+\;\Delta_{\mathrm{longitudinal}}^{2}+\Delta_{\mathrm{lateral}}^{2}}\;\;$$

(2) $$\mathrm{Vecto}r_{\mathrm{rotation}}=\sqrt{\Delta_{\mathrm{roll}}^{2}+\;\Delta_{\mathrm{pitch}}^{2}+\Delta_{\mathrm{yaw}}^{2}}$$

where $\Delta_{\mathrm{vertical}}$, $\Delta_{\mathrm{longitudinal}}$, $\Delta_{\mathrm{lateral}},\;\Delta_{\mathrm{roll}}$, $\Delta_{\mathrm{pitch}}$ and $\Delta_{\mathrm{yaw}}\;$are the shift detectability of EXTD_Thml, EXTD_Xray, and CBCT in vertical, longitudinal, lateral, roll, pitch, and yaw directions, respectively.

### 2.3 Statistical analyses

Statistical analyses were performed using SPSS Statistics version 17.0 (SPSS, Inc., Chicago, IL, USA) in this study. Wilcoxon signed‐ranks test was conducted to investigate if there were significant difference in the shift detection of EXTD_Thml, EXTD_Xray, and CBCT, where 0.05 was set to be the significance level (α) for rejecting the null hypothesis.

## 3 RESULTS

The differences in positional shift detected by EXTD_Thml and EXTD_Xray compared with CBCT in all 6 directions are summarized in Tables 1 and 2 for cranial and pelvis verification phantoms, respectively. The differences in translations and rotations reported by EXTD_Thml and EXTD_Xray against CBCT were less than 0.8 mm and 0.7° for both phantoms. Wilcoxon signed‐ranks test showed that the differences between EXTD_Thml and CBCT were significant in lateral, longitudinal, and yaw directions for both phantoms, while the difference between EXTD_Xray and CBCT were significant in lateral and longitudinal directions (*p* < 0.05).

**TABLE 1:** Differences of shift detected by EXTD_Thml and EXTD_Xray against cone‐beam computed tomography (CBCT) for 20 measurements in all six directions for cranial verification phantom

	EXTD_Thml			EXTD_Xray		
Direction	Average ± 1 SD	Range	*p*‐Value*	Average ± 1 SD	Range	*p*‐Value*
Vertical $\Delta_{\mathrm{vertical}}$ (mm)	0.23 ± 0.19	−0.6, 0.7	0.447	0.26 ± 0.19	−0.7, 0.6	0.567
Longitudinal $\Delta_{\mathrm{longitudinal}}$ (mm)	0.40 ± 0.26	−0.8, 0.3	0.001**	0.39 ± 0.28	−0.8, 0.4	0.001**
Lateral $\Delta_{\mathrm{lateral}}$ (mm)	0.34 ± 0.26	−0.9, 0.2	0.002**	0.37 ± 0.26	−0.9, 0.2	0.003**
Vector_translation_ (mm)	0.57 ± 0.41			0.60 ± 0.43		
Roll $\Delta_{\mathrm{roll}}$ (°)	0.11 ± 0.09	−0.2, 0.3	0.640	0.06 ± 0.08	−0.2, 0.2	0.623
Pitch $\Delta_{\mathrm{pitch}}$ (°)	0.11 ± 0.08	−0.3, 0.1	0.003**	0.09 ± 0.14	−0.6, 0.1	0.223
Yaw $\Delta_{\mathrm{yaw}}$ (°)	0.11 ± 0.10	−0.2, 0.4	0.032**	0.07 ± 0.09	–0.3, 0.2	0.874
Vector_rotation_ (°)	0.19 ± 0.16			0.13 ± 0.18		

* The *p*‐value was calculated using Wilcoxon signed‐ranks test.

** The *p*‐value has a statistical significance of difference (*p* < 0.05).

**TABLE 2:** Differences of shift detected by EXTD_Thml and EXTD_Xray against cone‐beam computed tomography (CBCT) for 20 measurements in all six directions for pelvis verification phantom

	EXTD_Thml			EXTD_Xray		
Direction	Average ± 1 SD	Range	*p*‐Value*	Average ± 1 SD	Range	*p*‐Value*
Vertical $\Delta_{\mathrm{vertical}}$ (mm)	0.24 ± 0.17	−0.7, 0.4	0.965	0.34 ± 0.17	−0.8, 0.5	0.027**
Longitudinal $\Delta_{\mathrm{longitudinal}}$ (mm)	0.47 ± 0.32	−1.0, 0.2	0.001**	0.59 ± 0.41	−1.3, 0.1	<0.001**
Lateral $\Delta_{\mathrm{lateral}}$ (mm)	0.39 ± 0.18	−0.8, 0.4	0.001**	0.34 ± 0.21	−0.8, 0.3	0.001**
Vector_translation_ (mm)	0.66 ± 0.40			0.76 ± 0.49		
Roll $\Delta_{\mathrm{roll}}$ (°)	0.13 ± 0.19	−0.2, 0.9	0.064	0.23 ± 0.25	−0.5, 1.1	0.158
Pitch $\Delta_{\mathrm{pitch}}$ (°)	0.11 ± 0.07	−0.2, 0.2	0.829	0.51 ± 0.31	−0.3, 1.3	0.001**
Yaw $\Delta_{\mathrm{yaw}}$ (°)	0.09 ± 0.08	−0.1, 0.2	0.012**	0.31 ± 0.24	−0.6, 1.1	0.012**
Vector_rotation_ (°)	0.19 ± 0.22			0.65 ± 0.46			

* The *p*‐value was calculated using Wilcoxon signed ranks test.

** The *p*‐value has a statistical significance of difference (*p* < 0.05).

More specifically, the translational difference between EXTD_Thml and CBCT was 0.57 ± 0.41 mm and 0.66 ± 0.40 mm for cranial and pelvis phantom, respectively, while it was 0.60 ± 0.43 mm and 0.76 ± 0.49 mm between EXTD_Xray and CBCT, respectively. For rotational movement, the difference between EXTD_Thml and CBCT was 0.19 ± 0.16° and 0.19 ± 0.22° for cranial and pelvis phantom, respectively, while it was 0.13 ± 0.18° and 0.65 ± 0.46° between EXTD_Xray and CBCT, respectively. It was found that the rotational difference of EXTD_Xray against CBCT was comparatively larger in the pelvis phantom than in the cranial phantom.

As shown in Table 3, the translational and rotational differences between EXTD_Thml and EXTD_Xray for both phantoms were less than 0.4 mm and 0.6°, respectively, with larger difference being observed for the rotational direction of pelvis phantom (0.58 ± 0.37°) compared with cranial phantom (0.18 ± 0.19°). Wilcoxon signed‐ranks test showed that there were significant differences between EXTD_Thml and EXTD_Xray for the pitch and yaw directions for both phantoms (*p* < 0.05).

**TABLE 3:** Differences of shift detected by EXTD_Thml and EXTD_Xray for 20 measurements in all six directions for cranial and pelvis verification phantoms

	Cranial phantom			Pelvis phantom		
Direction	Average ± 1 SD	Range	*p*‐Value*	Average ± 1 SD	Range	*p*‐Value*
Vertical $\Delta_{\mathrm{vertical}}$ (mm)	0.10 ± 0.09	−0.2, 0.4	0.506	0.16 ± 0.14	−0.2, 0.4	0.003**
Longitudinal $\Delta_{\mathrm{longitudinal}}$ (mm)	0.11 ± 0.11	‐0.3, 0.3	0.872	0.19 ± 0.16	−0.3, 0.6	0.012**
Lateral $\Delta_{\mathrm{lateral}}$ (mm)	0.05 ± 0.06	−0.1, 0.2	0.564	0.16 ± 0.14	−0.5, 0.4	0.618
Vector_translation_ (mm)	0.16 ± 0.15			0.30 ± 0.25		
Roll $\Delta_{\mathrm{roll}}$ (°)	0.10 ± 0.11	−0.2, 0.4	0.260	0.15 ± 0.11	−0.4, 0.4	0.662
Pitch $\Delta_{\mathrm{pitch}}$ (°)	0.12 ± 0.14	−0.3, 0.6	0.044**	0.50 ± 0.28	−1.1, 0.5	0.001**
Yaw $\Delta_{\mathrm{yaw}}$ (°)	0.09 ± 0.07	−0.1, 0.2	0.006**	0.26 ± 0.22	−0.9, 0.6	0.045**
Vector_rotation_ (°)	0.18 ± 0.19			0.58 ± 0.37		

* The *p*‐value was calculated using Wilcoxon signed ranks test.

** The *p*‐value has a statistical significance of difference (*p* < 0.05).

## 4 DISCUSSION

This study evaluated both the performance of EXTD_Thml and EXTD_Xray for shift detection compared with CBCT using anthropomorphic cranial and pelvis phantoms. The differences of both EXTD_Thml and EXTD_Xray against CBCT were found to be less than 0.8 mm and 0.7° in translations and rotations for both of the phantoms. It demonstrated that EXTD_Thml and EXTD_Xray could provide comparable accuracy for shift detection as CBCT on stationary phantoms.

The average difference between EXTD_Thml and CBCT in this study was 0.57 ± 0.41 mm for translational direction and 0.19 ± 0.16° for rotational direction using the cranial phantom, with a maximum rotational difference of 0.4°. These results were compared with other optical surface tracking systems conducted by Mancosu et al. on OSMS system (Varian) (0.6 ± 0.3 mm and 0.3° as the maximum rotational difference) and Peng et al. on AlignRT system (Vision RT) (0.9 ± 0.5 mm and 1.3° as maximum rotational difference) using a cranial phantom.^24^, ^25^ Also, our results were comparable with the previous study on Catalyst HD (C‐RAD Positioning AB, Uppsala, Sweden) using pelvis phantom. Kojima et al. reported that the accuracy for translation and rotation was less than 1 mm and 1°, with the worst in the longitudinal direction (0.9 ± 0.8 mm).^26^ Our result also showed that the difference of EXTD_Thml and CBCT in the longitudinal direction (0.47 ± 0.32 mm) was comparatively larger than the vertical (0.24 ± 0.17 mm) and lateral (0.39 ± 0.18 mm) directions. It was believed that the larger difference along the longitudinal direction was due to the cylindrically symmetric surface of the pelvis phantom, leading to inferior surface registration.^26^ Also, the resolution of CT images that was determined by slice thickness might induce higher uncertainty on shift detection in the longitudinal direction as reported by Gevaert et al.^27^ and Yan et al.^28^

Apart from EXTD_Thml, the current study also evaluated the accuracy of shift detection by EXTD_Xray against CBCT. The average differences between EXTD_Xray and CBCT for translation and rotation in this study were 0.76 ± 0.49 mm and 0.65 ± 0.46°, respectively, using the pelvis phantom. These results were comparable with the previous study on the setup discrepancies measured with the ExacTrac X‐ray 6D (previous version of EXTD) and CBCT on an anthropomorphic phantom containing spine structure by Chang et al. (<1.0 mm and <1.0 degree).^12^, ^29^ Also, Ma et al. conducted a study on the residual setup errors between ExacTrac X‐ray 6D and CBCT using a cranial phantom and reported that the errors were less than 0.5 mm and 0.2° for translational and rotational directions, respectively.^30^, ^31^ These findings were comparable with our results, in which the average differences between EXTD_Xray and CBCT using cranial phantom were 0.60 ± 0.43 mm for translational direction and 0.13 ± 0.18° for rotational direction.

The shift reported in the rotational direction by EXTD_Xray against CBCT was comparatively larger in pelvis phantom compared with cranial phantom. The possible reason was the poorer visibility of anatomical structures in stereoscopic X‐ray images for a thicker pelvis region compared with the cranial region. The acquired 2D planar X‐ray images for the pelvis region might not be always optimal for image registration with DRRs. On the contrary, CBCT can offer volumetric images with clear details of both bony landmarks and soft tissues for image registration. As a result, larger difference between EXTD_Xray against CBCT was observed for pelvis phantom.^12^

One of the major limitations of this study was the focus of phantom‐based measurements, in which the patient movements and anatomical changes during treatments were not considered. Further studies, therefore, could be conducted on patients to further examine the beneficial effect of optical surface and thermal tracking in real clinical scenarios and its ability to monitor the intrafractional movements during treatments.

## 5 CONCLUSION

This study demonstrated that the EXTD system with thermal mapping ability could provide comparable accuracy for shift detection when compared with CBCT on both cranial and pelvis phantoms.

## CONFLICT OF INTEREST

The authors declare that they have no conflict of interest.

## AUTHOR CONTRIBUTIONS

Vivian U. Y. Chow was responsible for research design, literature review, data collection, data analysis and manuscript writing. Michael L. M. Cheung was responsible for research design, data collection, data analysis and manuscript editing. Monica W. K. Kan was responsible for research design and manuscript editing. Anthony T. C. Chan was responsible for research guidance and supervision.
